# Dystonia and Parkinsonism in COA7-related disorders: expanding the phenotypic spectrum

**DOI:** 10.1007/s00415-023-11998-3

**Published:** 2023-09-26

**Authors:** Yujiro Higuchi, Masahiro Ando, Fumikazu Kojima, Junhui Yuan, Akihiro Hashiguchi, Akiko Yoshimura, Yu Hiramatsu, Satoshi Nozuma, Shinobu Fukumura, Hiroyuki Yahikozawa, Erika Abe, Itaru Toyoshima, Masashiro Sugawara, Yuji Okamoto, Eiji Matsuura, Hiroshi Takashima

**Affiliations:** 1 Department of Neurology and Geriatrics, Kagoshima University Graduate School of Medical and Dental Sciences, 8-35-1 Sakuragaoka, Kagoshima City, Kagoshima 890-8520 Japan; 2 Department of Pediatrics, Sapporo Medical University School of Medicine, Sapporo, Japan; 3 Yahikozawa Internal Medicine and Neurology Clinic, Nagano, Japan; 4 Department of Neurology, National Hospital Organization Akita National Hospital, Yurihonjo, Japan; 5 Department of Neurology, Akita University Graduate School of Medicine, Akita, Japan; 6 Department of Physical Therapy, School of Health Sciences, Faculty of Medicine, Kagoshima University, Kagoshima, Japan

**Keywords:** *COA7*, Spinocerebellar ataxia with axonal neuropathy, Mitochondrial disease, Parkinsonism, Dystonia

## Abstract

**Background and objective:**

Biallelic mutations in the *COA7* gene have been associated with spinocerebellar ataxia with axonal neuropathy type 3 (SCAN3), and a notable clinical diversity has been observed. We aim to identify the genetic and phenotypic spectrum of *COA7*-related disorders.

**Methods:**

We conducted comprehensive genetic analyses on the *COA7* gene within a large group of Japanese patients clinically diagnosed with inherited peripheral neuropathy or cerebellar ataxia.

**Results:**

In addition to our original report, which involved four patients until 2018, we identified biallelic variants of the *COA7* gene in another three unrelated patients, and the variants were c.17A > G (p.D6G), c.115C > T (p.R39W), and c.449G > A (p.C150Y; novel). Patient 1 presented with an infantile-onset generalized dystonia without cerebellar ataxia. Despite experiencing an initial transient positive response to levodopa and deep brain stimulation, he became bedridden by the age of 19. Patient 2 presented with cerebellar ataxia, neuropathy, as well as parkinsonism, and showed a slight improvement upon levodopa administration. Dopamine transporter SPECT showed decreased uptake in the bilateral putamen in both patients. Patient 3 exhibited severe muscle weakness, respiratory failure, and feeding difficulties. A haplotype analysis of the mutation hotspot in Japan, c.17A > G (p.D6G), uncovered a common haplotype block.

**Conclusion:**

*COA7*-related disorders typically encompass a spectrum of conditions characterized by a variety of major (cerebellar ataxia and axonal polyneuropathy) and minor (leukoencephalopathy, dystonia, and parkinsonism) symptoms, but may also display a dystonia-predominant phenotype. We propose that *COA7* should be considered as a new causative gene for infancy-onset generalized dystonia, and *COA7* gene screening is recommended for patients with unexplained dysfunctions of the central and peripheral nervous systems.

**Supplementary Information:**

The online version contains supplementary material available at 10.1007/s00415-023-11998-3.

## Introduction

Mitochondrial disorders are a clinically and genetically heterogeneous group of progressive multisystem disorders caused by impaired mitochondrial function. The neuromuscular manifestations observed in affected patients (which may encompass encephalopathy, seizures, dementia, deafness, ophthalmoplegia, myopathy, ataxia, neuropathy, as well as pyramidal and extrapyramidal signs) are characterized by their diversity and progression over time. We have identified the cytochrome c oxidase assembly factor 7 (*COA7*) gene as a novel causative gene for spinocerebellar ataxia with axonal neuropathy type 3 (SCAN3) [1]. The *COA7* gene, also known as respiratory chain assembly factor 1 (*RESA1*), sel1 repeat-containing protein 1 (*SELRC1*), and *C1orf163*, encodes a protein localized within the mitochondria and is involved in the assembly and activity of the mitochondrial respiratory chain (MRC) complex [2, 3]. To date, only seven affected patients with biallelic variants in *COA7* have been reported worldwide [1, 3–5]. Patients with *COA7*-related disorders may develop varied degrees of cerebellar ataxia and axonal polyneuropathy, in combination with other symptoms or signs such as developmental regression, spasticity, cognitive dysfunction with leukoencephalopathy and spinal cord atrophy, and subclinical mitochondrial myopathy.

In this study, we included three additional patients with biallelic *COA7* variants. Among them, two patients presented with extrapyramidal symptoms such as dystonia, parkinsonism, or tremor, while the other patient exhibited severe muscle weakness, respiratory failure, and feeding difficulties. By reviewing a total of 10 phenotypes, including our own cases and previously reported ones, our findings further broaden the spectrum of symptoms associated with *COA7*-related disorders and raise awareness about the possibility of diverse phenotypes, particularly dystonia and parkinsonism in this rare condition.

## Materials and methods

### Patient selection and genetic analysis

**Fig. 1:**
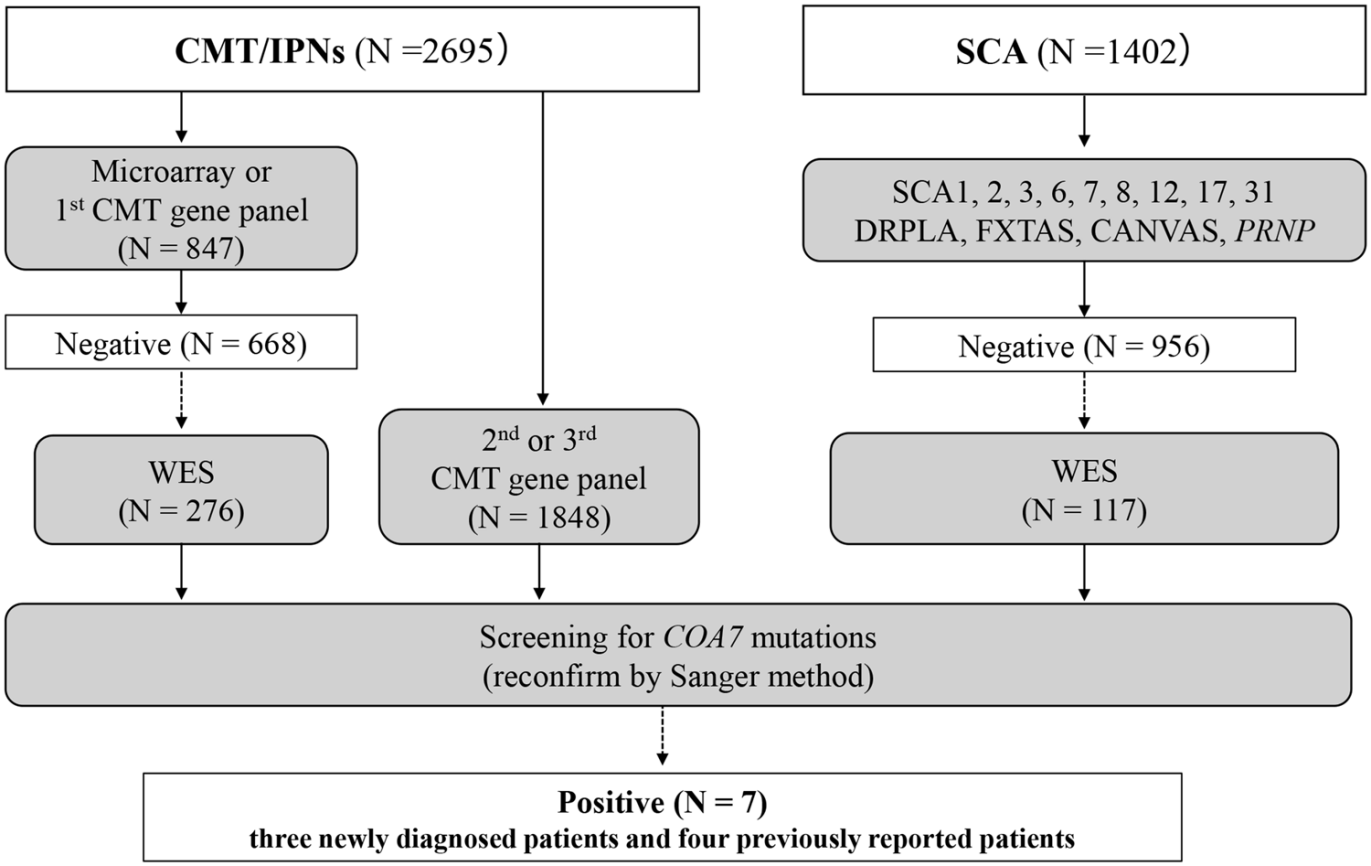
Genetic analysis flowchart of CMT/IPN and SCA in our study

Our institution serves as a genetic testing center in Japan, focusing on a group of inherited neurological diseases, including but not limited to inherited peripheral neuropathy (IPN), which is represented by Charcot–Marie–Tooth disease (CMT), and spinocerebellar ataxia (SCA) [6–8]. Between 2000 and 2022, a total of 4,097 patients (2,695 patients with CMT/IPN and 1,402 patients with SCA) were enrolled. We noted that a subset of patients may develop complex forms of CMT, where their neuropathy is accompanied by additional features such as cerebellar ataxia, cognitive impairment, spasticity, extrapyramidal tract signs, dystonia, and deafness. Figure 1 shows an overview of the patient selection process and genetic analysis workflow conducted in this study.

In the case series of CMT/IPN patients, we performed genetic analyses using DNA microarray, next-generation sequencing (NGS)-based CMT gene panel sequencing (three versions), or whole-exome sequencing (WES), as previously described [8, 9]. The *COA7* gene was not involved in the gene panels utilized for DNA microarray or our initial version of the NGS gene panel; thus, 276 out of the 668 mutation-negative CMT/IPN patients were processed to WES, enabling their *COA7* gene analysis to be available. Furthermore, a total of 1843 patients with CMT/IPN underwent *COA7* gene screening using either the second or third NGS gene panel. Gene lists of all three NGS gene panels are shown in Supplemental Table 1.

In a case series of patients with SCA, we screened for *COA7* variants using WES in 117 patients who tested negative for repeat expansions associated with spinocerebellar ataxias (SCA1, SCA2, SCA3, SCA6, SCA7, SCA8, SCA12, SCA17, SCA31, FXTAS, CANVAS, and DRPLA), as well as a Pro102Leu mutation in the *PRNP* gene, as previously described [10, 11].

We evaluated the identified variants according to American College of Medical Genetics and Genomics (ACMG) standards and guidelines [12].

### Haplotype analysis

Between now and our previous report, we have identified five unrelated patients carrying a recurring variant of the *COA7* gene (c.17A > G). To determine whether this mutation occurred from independent mutational events or common ancestry, we performed a single-nucleotide variant (SNV)-based haplotype analysis, utilizing 13 SNVs flanking the *COA7* gene, which were obtained from the WES data.

### In silico structural analyses and genetic tolerance analyses

To evaluate the effect of a novel variant, c.449G > A (p.Cys150Tyr), we conducted protein structure analyses using computational methods. Briefly, the three-dimensional (3D) structure of human COA7 (UniProt: Q96BR5) was acquired from the Protein Data Bank (PDB) (https://www.rcsb.org/pdb/; 7MQZ). In the 7MQZ protein structure, the steric structures of amino acid residues 10–218 (231 residues in total length) of human COA7 have been determined by X-ray crystallography [13]. The structural model of the C150Y mutant was constructed using SWISS-MODEL based on the structure of wild-type COA7 (7MQZ). Superposition and visualization of the wild-type and mutant 3D structures were performed using the Waals software program (Altif Labs Inc., Tokyo, Japan). The amino acid residue numbering of human COA7 is based on UniProt. MetaDome (https://stuart.radboudumc.nl/metadome) was utilized to measure the genetic tolerance of the entire *COA7* gene.

## Results

### Genetic findings

**Fig. 2:**
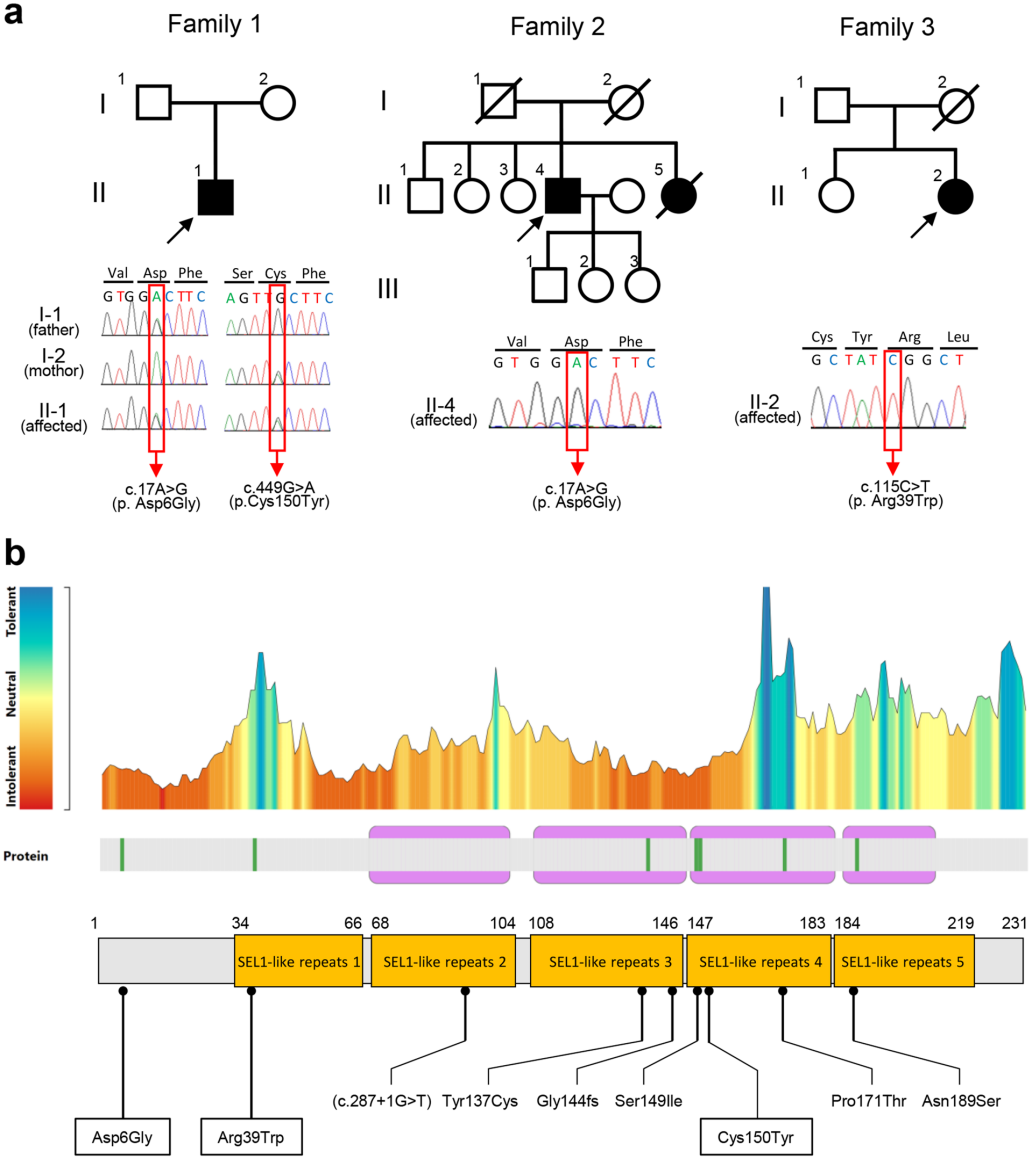
Genetic findings and mutation tolerance analysis. **a** Pedigree structure of three patients and segregation analysis (Patient 1). Squares represent males, and circles represent females. Filled symbols represent affected individuals, and open symbols represent unaffected individual. Oblique lines represent deceased family members. Black arrows indicate the proband (P1–P3). Red box indicates the mutation site. **b** Mutation tolerance landscape from MetaDome and location of *COA7* variants reported so far (variants detected in present study are highlighted in square)

We identified biallelic *COA7* variants from seven patients, four of whom had previously been described in 2018 [1] and three newly collected ones. These three new patients were identified from a case series of CMT/IPN patients. The biallelic variants detected in the *COA7* gene from three undescribed patients were: a compound heterozygous combination of a known pathogenic variant (c.17A > G, p.Asp6Gly) and a novel variant (c.449G > A, p.Cys150Tyr) in patient 1 and two known pathogenic homozygous variants, (c.17A > G, p.Asp6Gly) and (c.115C > T, p.Arg39Trp), in patients 2 and 3, respectively (Fig. 2A, Table 1).

**Table 1:** Clinical, neurological, and laboratory findings of patients with *COA7* biallelic variants

	This study			Our past cases[1]				Anabel et al. [3]	Ban et al. [4]	Ouchi et al. [5]
	Patient 1	Patient 2	Patient 3	Patient 4	Patient 5	Patient 6	Patient 7	Patient 8	Patient 9	Patient 10
Variants	c.17A > G (p.D6G) c. 449G > A (p.C150Y)	c.17A > G (p.D6G)	c.115C > T (p.R39W)	c.17A > G (p.D6G)	c.115C > T (p.R39W) exon 2 deletion	c.17A > G (p.D6G) c.446G > T (p.S149I)	c.17A > G (p.D6G) c.430delG (p.G144fs)	c.410A > G (p.Y137C) c.287 + 1G > T	c.511G > A (p.A171T) c.566A > G (p.N189S)	c.17A > G (p.D6G)
Gender/Age, yr	M/19	M/81	F/46	M/63	F/21	M/28	M/27	F/19	F/2	M/60
Onset age, yr	1	50	0	< 5	4	15	< 5	1	3 months	30
Clinical features										
Polyneuropathy	+	+	+	+	+	+	+	+	−	+
Cerebellar ataxia	−	+	+	+	+	+	+	+	−	+
Cognitive impairment	−	−	−	−	+	−	−	+	−	−
Extrapyramidal symptom	+ (dystonia)	+ (parkinsonism)	−	−	−	−	−	−	+ (dystonia)	+ (parkinsonism, dystonia)
Tremor	−	+	−	−	−	−	−	−	−	−
Pyramidal symptom	+	−	−	−	−	−	−	−	+	−
Neuroimaging findings										
Cerebellar atrophy	−	+	+	+	+	+	+	−	−	+
Cerebral atrophy	−	+	−	−	+	−	−	−	+	−
Brainstem atrophy	−	+	+	−	−	−	−	−	NA	−
Spinal cord atrophy	NA	+	+	−	NA	+	−	+	+	NA
Cerebral white matter lesions	−	+	+	−	+	−	+	+	+	−
Other	Abnormal DAT-SPECT	Abnormal DAT-SPECT	Dysphagia (PEG), vocal cord paralysis, respiratory failure (TPPV)	None	None	None	None	Cavitating leukodystrophy of the brain	Mild cardiomyopathy	Abnormal DAT-SPECT
Hyper-CKemia	NA	+	NA	+	+	+	+	NA	+	−
Nerve Conduction Study	Axonal (sensory)	Axonal	Axonal	Axonal	Axonal	Axonal	Axonal	NA	NA	Mixed axonal demyelinating pattern

*DAT-SPECT* dopamine transporter single-photon emission computed tomography, *NA* not available, *PEG* percutaneous endoscopic gastrostomy, *TPPV* tracheostomy positive pressure ventilation

In our previous study, regarding the Asp6Gly and Arg39Trp variants, we demonstrated activity reduction of either the MRC enzyme complex I or complex IV, using patient-derived skin fibroblasts or muscle tissues [1]. Thereafter, both variants were further confirmed to be pathogenic via the cellular rescue assay by Formosa et al. [13]. Taken together, both variants were classified as pathogenic according to the ACMG guideline.

On the other hand, the novel variant, c.449G > A (p.Cys150Tyr), of patient 1 was not found in multiple public databases (Supplemental Table 2) or our in-house database (ACMG criteria; PM2). Compound heterozygous variants were found to be co-segregated with the disease in this family (ACMG criteria; PP1) and identified *in trans* with a known pathogenic variant (ACMG criteria; PM3) (Fig. 2A). Cys150 residues are highly conserved throughout multiple species (Supplemental Fig. 1). MetaDome analyses revealed that the c.449G > A variant was located on the SEL1-like repeat 4 region, which is highly intolerant to genomic variants (Fig. 2B). Computational analyses using multiple tools (SIFT, Polyphen2, PROVEAN, Mutation Assessor, and CADD) indicate that this variant has damaging effects (ACMG criteria; PP3) (Supplemental Table 2). Altogether, we classified these variants as likely pathogenic according to the ACMG guideline.

### Clinical features of three newly identified patients with COA7 biallelic variants

The genetic, clinical, and electrophysiological findings of three newly identified patients, along with a literature review of previously described cases carrying biallelic *COA7* variants, are summarized in Table 1 and Supplemental Table 2.

### Patient 1 (19 yo, male)

**Fig. 3:**
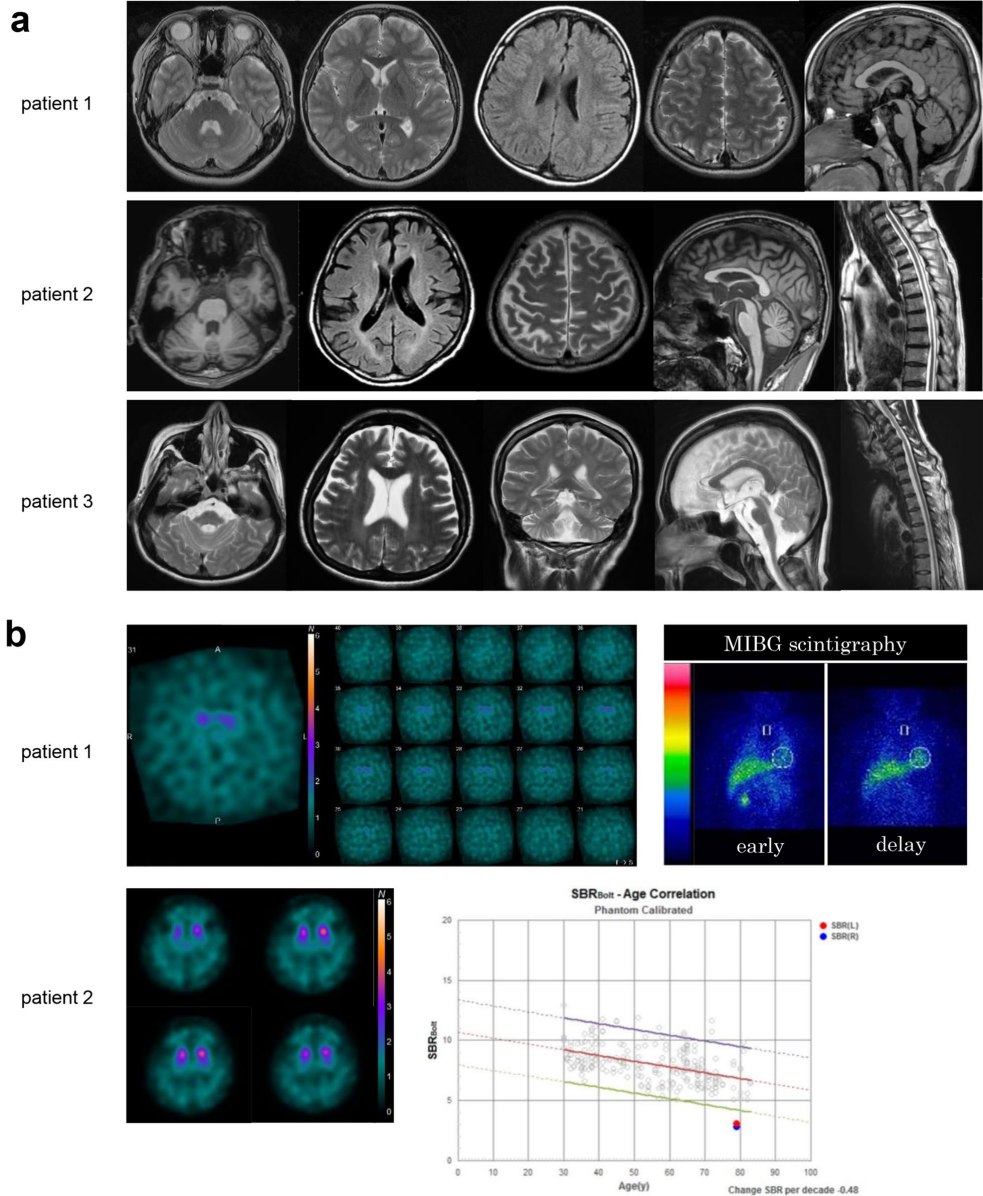
MRI findings, dopamine transporter SPECT, and MIBG scintigraphy. **a** Brain MRI of Patient 1 at the age of 11 years shows no obvious atrophy or significant white matter abnormalities in the cerebrum, brainstem, or cerebellum on T1-weighted, T2-weighted, and fluid-attenuated inversion recovery (FLAIR) images. Axial MRI images from Patient 2 show atrophy of the cerebellum, brainstem, spinal cord, and cerebral cortex, with a slightly high-intensity lesions in the bilateral cerebral white matter on FLAIR image. MRI of Patient 3 shows obvious atrophy of cerebrum, brainstem, and spinal cord, with mild bilateral hyperintensity in the periventricular white matter on T2-weighted image. **b** DAT scans of Patient 1 at the age of 11 years indicate bilateral decreased dopamine transporter uptake. MIBG scintigraphy shows normal uptake. DAT scans of Patient 2 at the age of 79 years show bilateral decreased dopamine transporter uptake

Patient 1, a 19-year-old male, was born to healthy non-consanguineous parents and had no family history of similar symptoms. He was referred to the local child development center due to developmental delays. He achieved the ability to sit unassisted at 8–9 months, pull himself up to standing at 12 months, and began walking at 15 months of age. At the age of 2 years, he started experiencing involuntary movements in his toes, and bilateral spastic paralysis emerged when he was 2 years and 9 months of age. At the age of 3 years and 10 months, he was diagnosed with dystonia, which was characterized by generalized tension and postural abnormalities. He became unable to walk by the age of 5 years. Although levodopa administration provided only slight and temporary improvement, his symptoms progressively worsened over time, eventually becoming refractory. Neurological examinations at six years of age revealed truncal and limb dystonia with a significant reduction of muscle extensibility and increased resistance to passive movement. Deep tendon reflexes were increased. No apparent signs of cerebellar ataxia, sensory disturbance, or intellectual disability were observed. Subsequently, he developed severe painful dystonia, accompanied by dysarthria and dysphagia, and was hospitalized at the age of 12. To reassess the effectiveness of levodopa, a temporary discontinuation of levodopa was implemented, which was subsequently followed by its resumption. Notably, there was a significant improvement in generalized dystonia after reintroduction of levodopa, with the reduction of Burke–Fahn–Marsden Dystonia Rating Scale-Movement scale (BFMDRS-M) [14] scores from 86 to 56 points, and the Unified Dystonia Rating Scale (UDRS) [15] scores from 61.5 to 55.5 points. Subsequently, he underwent bilateral deep brain stimulation (DBS) targeting the globus pallidus internus, and a further improvement was observed in dysarthria and dystonia affecting his larynx, neck, trunk, and upper limbs. And his BFMDRS-M scores were found decreased from 56 to 37 points, and the UDRS scores decreased from 55.5 to 43.5 points. Unfortunately, however, his dystonia in the neck, truck, and four limbs gradually deteriorated year by year, and by the age of 19, he became bedridden and requires full assistance for dressing and toileting. Although his brain MRI showed no apparent atrophy or signal abnormalities (Fig. 3A), dopamine transporter SPECT (DAT-SPECT) revealed decreased uptake in the bilateral putamen (specific binding ratios [SBRs]: right, 3.57; left, 2.23 [prediction intervals 95% lower limit to the mean SBR in the 70–79 age group, 4.42]; asymmetry index, 46.3%; Fig. 3B). Cardiac ^123^I-meta-iodobenzylguanidine myocardial scintigraphy (MIBG scintigraphy) revealed normal uptake (heart/mediastinum ratio: early, 2.6; delayed, 2.43 [lower limit of our institute, 2.2]; and washout rate, 17.1% [upper limit, 22%]; Fig. 3B). Motor nerve conduction studies (NCSs) did not reveal any apparent abnormalities, whereas sensory NCSs showed absent sensory conduction in the median, ulnar, and sural nerves, indicating sensory polyneuropathy.

### Patient 2 (81 yo, male)

Patient 2 was an 81-year-old male who reported experiencing leg discomfort (difficulty in movement) since the age of 15 years, although he did not encounter issues with activities such as climbing/descending stairs or running. At the age of 50 years, he developed dysesthesia in both distal lower extremities and gait abnormalities. Due to a slowly progressive unsteady gait, he was hospitalized at 60 years of age. His neurological examination revealed predominant distal muscle atrophy/weakness, and a complete loss of sensation across all sensory modalities in the lower limbs. Ankle reflexes were absent. This patient also exhibited tremors in the limbs, trunk, and mouth, along with rigidity in the limbs, trunk, and neck, accompanied by slurred speech. His tremor was characterized by gross and irregular pattern with a combination of resting, postural, and action tremors. His gait was characterized by a wide-based and short-stepped pattern, along with a positive Romberg’s sign, which indicated a combination of parkinsonian, cerebellar, and sensory ataxic features. Notably, he had no evident cognitive impairment. Levodopa administration resulted in improvements in limb and mouth tremors, limb rigidity, and his stepping gait. MRI scans revealed cerebellar atrophy and mild atrophy in the cerebral and spinal cord regions (Fig. 3A). DAT-SPECT revealed decreased uptake in the bilateral putamen (SBRs: right, 2.83; left, 3.10) (Fig. 3B). NCSs revealed absent motor and sensory responses in the lower limbs, suggesting an axonal type of motor and sensory neuropathy. The conduction velocities in the median and ulnar nerves were within normal limits (Supplementary Table 3). Sural nerve biopsy with histopathology revealed a remarkable decrease in the density of large and medium myelinated fibers: thin myelin sheaths and clusters of myelinated fibers. These findings are indicative of chronic axonal degeneration. No inflammatory cells or onion-bulb formations were observed. His deceased younger sister was suspected of exhibiting a similar phenotype, whereas no similar symptoms were identified in his parents (Fig. 2A).

### Patient 3 (46 yo, female)

Patient 3, a 46-year-old female, was born to consanguineous parents. There was no similar clinical symptom identified in any of her family members, and her mother had already died of a cerebral hemorrhage at the age of 64 (Fig. 2A). This patient exhibited gross motor developmental delay since infancy. She achieved turning over, sitting up, and crawling by 12 months of age, standing at 14 months, and walking at 18 months. During early childhood, she experienced an unsteady gait and a tendency to fall. At the age of 8 years, she was diagnosed with spinal muscular atrophy and underwent surgical tendon lengthening. By the age of 13 years, she required a wheelchair for mobility. When she was examined by a neurologist at age 19 years, she displayed severe muscle atrophy/weakness that predominantly affected the distal regions, sensory loss in her lower limbs, ataxia, and absent ankle reflexes. She also developed a mild intellectual disability, manifesting in reduced Adult Intelligence Scale-Revised (WAIS-R) scores, including a verbal score of 78, a performance score of 65, and a full-scale IQ of 68. During her 20 s, she developed hoarseness (vocal cord paralysis) and dysphagia and underwent a percutaneous endoscopic gastrostomy. In her 30 s, she experienced progressive respiratory muscle paralysis, resulting in a significant decline in her vital capacity requiring a tracheostomy and invasive ventilation to support her breathing. MRI scans revealed evident atrophy in the cerebellum, brainstem, and spinal cord, as well as mild white matter lesions (Fig. 3A). NCSs at 19 years of age indicated a severe, active, length-dependent axonal motor-sensory peripheral neuropathy. Motor and sensory responses were absent in her lower limbs, and the conduction velocity in her median nerve was normal. Needle electromyography (EMG) revealed neurogenic changes characterized by long durations and large amplitudes of motor unit action potentials (MUPs), with a chronic loss of motor units. Laboratory studies revealed normal serum creatine kinase levels.

### SNV-based homologous haplotype around COA7

**Fig. 4:**
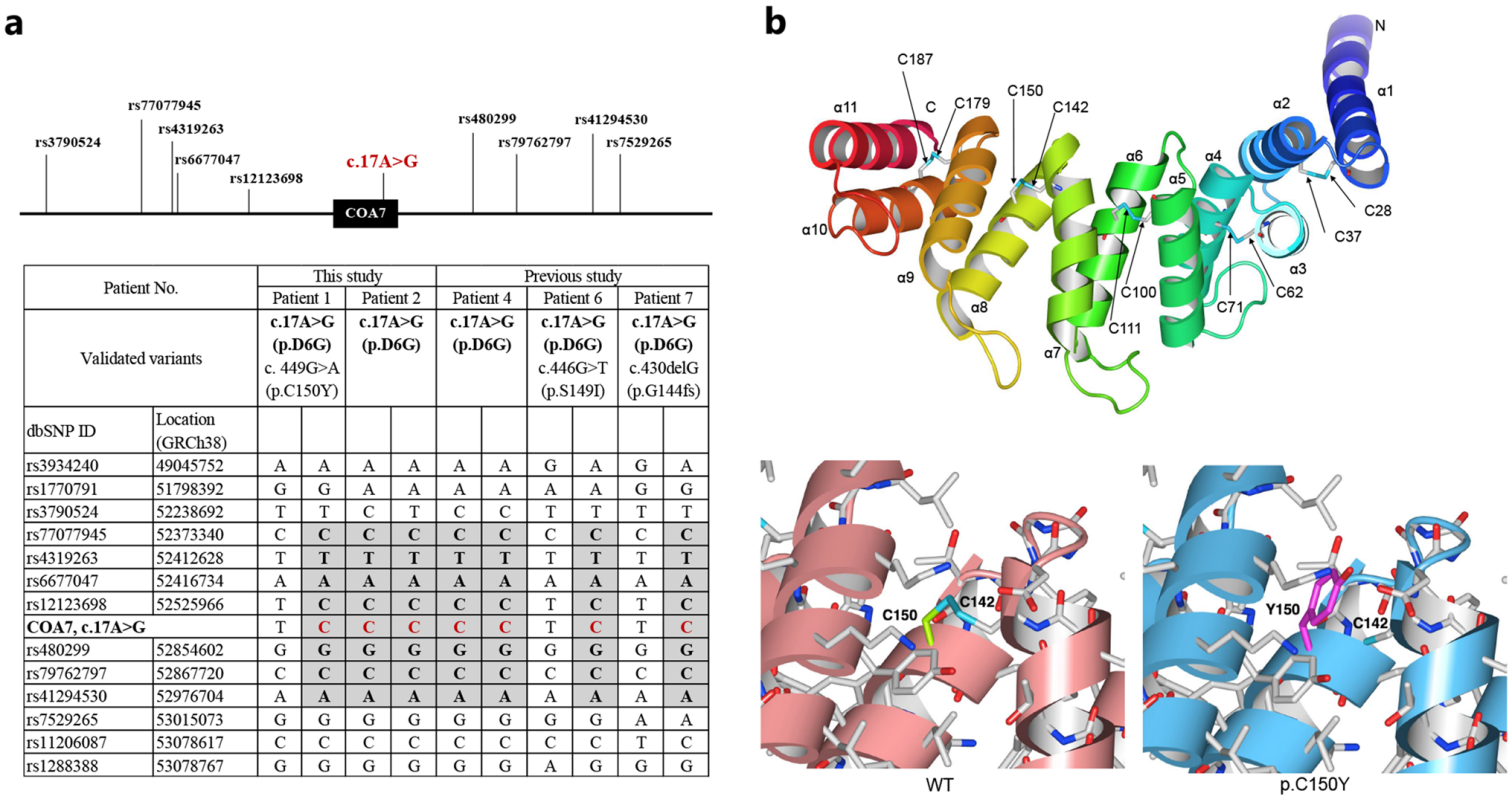
Haplotype analysis and 3D structures of wild-type COA7 and the p.Cys150Tyr mutant. **a** Haplotype analysis shows an identical ~ 0.6 Mb haplotype block (from rs77077945 to rs41294530), spanning the hotspot c.17A > G variant of five proband patients. **b** In the upper right panel, the 3D structure of COA7 (PDB ID: 7MQZ) is shown in ribbon format with a blue-to-red gradient from the N-terminus to the C-terminus. The cysteine residues involved in disulfide bond formation are depicted in stick format, with carbon atoms in gray, oxygen atoms in red, nitrogen atoms in blue, and sulfur atoms in cyan. In the lower right panel, each amino acid residue is shown in stick representation. It is predicted that the disulfide bond between Cys150 and Cys142 is lost due to the substitution of Cys150 to Tyr150

The haplotype was inferred by analyzing genomic variants obtained from WES data of five patients who shared a recurring *COA7* variant (c.17A > G). Haplotype analyses using multiple SNV markers identified an identical haplotype block of ~ 0.6 Mb in size (from rs77077945 to rs41294530). This block spanned the c.17A > G variant and was observed in all five probands, suggesting a founder effect (Fig. 4a).

### The p.Cys150Tyr mutant affects the structure of human COA7

To investigate the structural impact of the novel mutant, p.Cys150Tyr in COA7, we constructed a structural model of the mutant protein using the wild-type COA7 structure (7MQZ) as a reference. The generated structural model using SWISS-MODEL met the reliability criteria and obtained a good score, as indicated by a QMEANDisCo Global score of > 0.5 (Supplemental Fig. 2).

Human COA7 consists of 11 alpha helices (α1-α11), which include 5 α/α repeats and a C-terminal alpha helix. Ten of the 13 cysteine residues in COA7 are involved in the formation of disulfide bonds (Cys28-Cys37, Cys62-Cys71, Cys100-Cys111, Cys142-Cys150, Cys179-Cys187) between alpha helices in the α/α repeats. Cys150 is located on the eighth alpha helix within the fourth α/α repeat and forms a disulfide bond with Cys142 in the seventh alpha helix of the same repeat (repeat 4) (Fig. 4b and Supplemental Fig. 3). Figure 4b shows the 3D structures of wild-type COA7 and the p.Cys150Tyr mutant and illustrates the impact of the variant on the nearby Cys150 residue that forms a disulfide bond with Cys142 in the wild type. The substitution of Tyr for the Cys residue at codon 150 is expected to eliminate the thiol group (–SH) located in the side chain of the Cys residue, thereby hindering the formation of disulfide bonds. Consequently, the missense variant, p.Cys150Tyr, is suggested to destabilize the three-dimensional structure of COA7 by leading to the loss of this disulfide bond between Cys150 and Cys142.

## Discussion

In this study, we present three additional Japanese patients with biallelic variants in the *COA7* gene. These patients exhibit intriguing clinical phenotypes, including extrapyramidal symptoms such as dystonia and parkinsonism, as well as a severe form of the condition, characterized by vocal cord paralysis and respiratory failure requiring assisted ventilation.

To date, seven patients with *COA7* biallelic variants have been reported in the literature, with an expanding phenotypic spectrum. The clinical phenotypes and genotypes of all the published patients carrying biallelic variants in *COA7* (together with our patients) are listed in Table 1. A patient initially reported by Martinez Lyons et al. in 2016 presented with an early-onset, progressive neurological disorder characterized by severe ataxia, peripheral neuropathy, and mild cognitive impairment, accompanied by leukoencephalopathy and spinal cord atrophy [3]. In our subsequent report published in 2018, we described four additional patients who exhibited the characteristic neurological features of neuropathy and cerebellar ataxia, leading us to designate them as SCAN3. Some of these patients showed leukoencephalopathy or spinal cord atrophy on MRI scans, with variations in terms of onset, severity, and central nervous system involvement. Moreover, Rui Ban et al. reported Chinese patient with developmental regression, progressive spasticity, leukoencephalopathy, brain atrophy, and mild cardiomyopathy, without ataxia and neuropathy, having a considerably different phenotype and age of onset than previously reported patients. Intriguingly, a recent study found that a 60-year-old man with a homozygous *COA7* variant, c.17A > G (p.D6G), had adult-onset (> 30 years) progressive neurological impairments, characterized by cerebellar symptoms, sensorimotor neuropathy, and parkinsonism [5].

On the other hand, Patients 1 and 2 in this study exhibited extrapyramidal symptoms, with Patient 1 displaying a novel neurological sign, dystonia. Despite the difference in their ages of onset (1 year and 50 years for Patient 1 and Patient 2, respectively), both patients exhibited generalized hypertonia affecting the muscles of the limbs and trunk. Levodopa administration resulted in mild improvement, with Patient 1 experiencing more pronounced benefits. Additionally, patient 1 responded positively to DBS. Although MRI did not show any apparent abnormal signal or atrophy in the basal ganglia of both patients, DAT-SPECT revealed decreased uptake in the bilateral putamen. In Patient 1, who underwent MIBG scintigraphy, no reduction in uptake was observed, which is consistent with the findings reported by Ouichi et al. [5]. Patient 1 displayed a considerably different phenotype compared to previously documented patients with *COA7* variants in the following aspects: (1) Generalized dystonia emerged as the primary neurological symptom; (2) cerebellar ataxia and cerebellar atrophy were not observed, and (3) NCS showed a sensory neuropathy without involvement of motor nerves. Genetically, the *in trans* compound heterozygous combination of a known pathogenic variant (c.17A > G) and a novel likely pathogenic variant (c.449G > A) is responsible for the phenotype of Patient 1. WES did not identify any known pathogenic mutations in genes associated with hereditary dystonia or familial Parkinson’s disease, suggesting that *COA7* may be a new causative gene for infancy-onset generalized dystonia. Herein, we propose that this newly identified entity of generalized dystonia can be referred to as hereditary dystonia type 37 (DYT37). Further genetic screening in additional familial and sporadic dystonia cases is needed to refine and validate this phenotype.

COA7 expression data obtained from the Human Protein Atlas show that COA7 is highly expressed not only in the cerebellum but also in the cerebral cortex and basal ganglia (Supplemental Fig. 4). Previous studies have demonstrated that the nigrostriatal system is very vulnerable to mitochondrial dysfunction. Basal ganglia dysfunction is associated with various neurological features, such as dystonia, parkinsonism, and tremor, which are among the most prevalent movement disorders observed in patients with mitochondrial disorders [16–18]. Mitochondrial and nuclear genes associated with dystonia and parkinsonism are listed in Supplemental Table 4. Many of these genes are involved in the reduction of activity in MRC complex I or IV, as well as their assembly factors. In our previous functional analysis, we found that fibroblasts obtained from two patients with *COA7* biallelic mutations, namely Patient 5 and Patient 6 in Table 1, exhibited a deficiency in MRC complex IV [1]. Additionally, low complex I activity was observed in fibroblasts from one patient, namely Patient 4 in Table 1. Furthermore, in vitro cell culture studies and cellular rescue assays using CRISPR-Cas9-mediated gene editing have provided evidence of the potential role of COA7 in the assembly of MRC complexes I and IV [2, 13].

Although the exact molecular mechanism underlying the development of extrapyramidal signs due to COA7 dysfunction remains unclear, it is noteworthy that the COA7 protein is moderately expressed in the cerebral cortex and basal ganglia. Additionally, it is involved in the activity and expression of MRC complexes I and IV, indicating that *COA7* likely plays an important role in the cortico-basal ganglia circuit mechanism. There have been reports suggesting that mutations in the *SURF1* [19], *COX20* [20, 21], and *SCO2* [22] genes, which are involved in the assembly of COX along with COA7, can result in a decrease in the activity of MRC complex IV, leading to dystonia or parkinsonism. Mutations in the *NDUFA9* [23] and *NDUFAF6* [24, 25] genes, which are complex I assembly factors, have also been linked to dystonia. Interestingly, it has been recently demonstrated that COA7 is a heme-binding protein with disulfide reductase activity that transiently interacts with the copper metallochaperones SCO1 and SCO2, suggesting a role in the early stages of complex IV assembly [13]. Furthermore, similar to *COA7*-related disorders, mutations in the aforementioned genes exhibit a diversity of clinical manifestations, including cerebellar ataxia, neuropathy, or dementia. These observations indicate the possibility of a shared degenerative process among these diseases. These findings, along with the fact that the patients in both of our cases and another case reported by Ouchi et al. presented with extrapyramidal signs, support the hypothesis that biallelic variants of *COA7* are responsible for dystonia and parkinsonism.

**Fig. 5:**
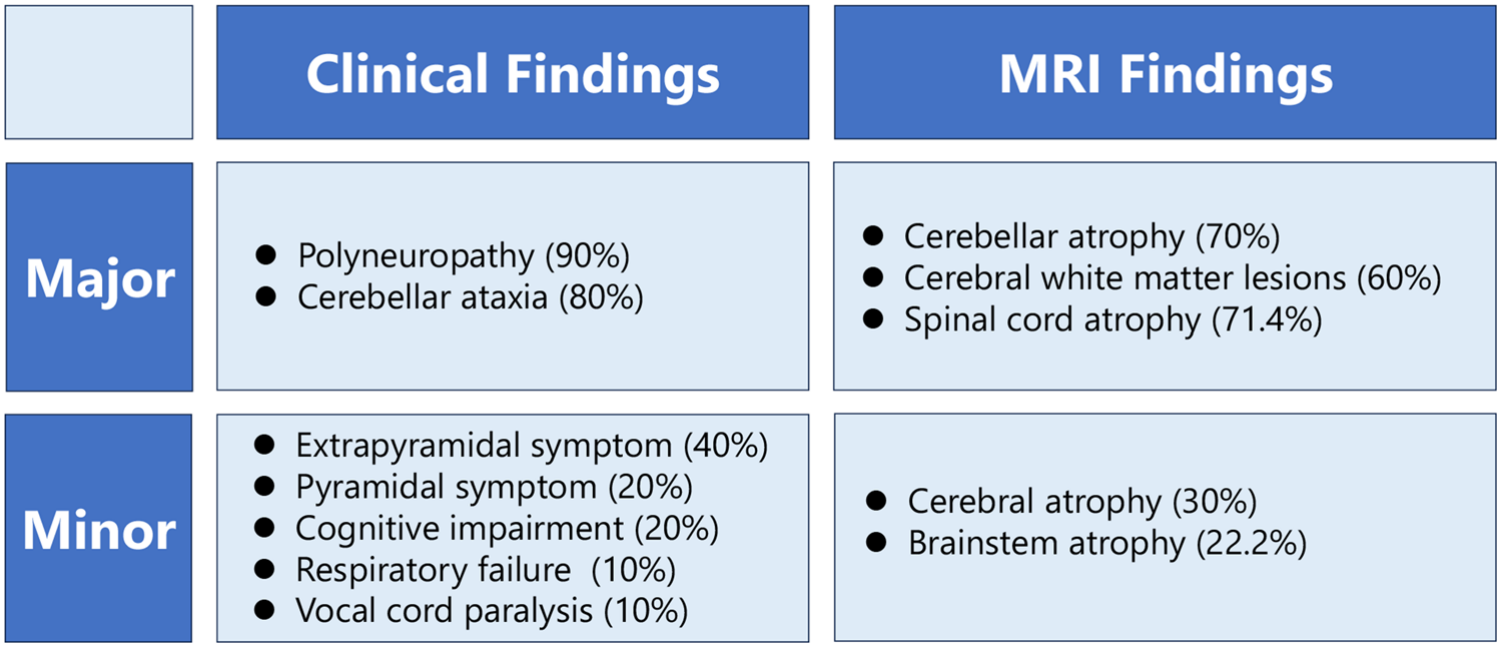
Phenotypic spectrum of *COA7*-related disorders. *COA7*-related disorders are characterized by a variety of major and minor symptoms and MRI findings

Patient 3 exhibited severe muscle atrophy and weakness affecting not only limb and trunk muscles but also respiratory muscles, resulting in respiratory failure. Multiple factors were considered to have contributed to the respiratory paralysis in this patient, including (1) severe axonal impairment, especially of the phrenic nerve; (2) myopathic impairment of respiratory muscles due to mitochondrial myopathy, and (3) airway narrowing caused by progressive vocal cord paralysis. NCS revealed axonal motor-sensory polyneuropathy, and neurogenic MUPs were observed via EMG. No hyper-CKemia was detected. Spina cord atrophy was also observed on MRI. Therefore, we believe that the main factor contributing to respiratory failure in this patient is severe axonal impairment, particularly affecting the phrenic nerve. This impairment is likely a result of peripheral motor neuropathy and/or spinal motor neuron degeneration. However, further histopathological and electrophysiological investigations are required to elucidate the molecular mechanisms underlying respiratory failure in this patient. Figure 5 summarizes the characteristics of major symptoms, minor symptoms, and MRI findings of *COA7*-related disorders based on current and previous reports.

## Conclusion

In summary, our study has broadened the phenotypic spectrum of *COA7*-related disorders, typically characterized by cerebellar ataxia and neuropathy as major neurological manifestations, accompanied by minor phenotypes, such as dystonia, parkinsonism, intellectual disability, spasticity, and respiratory paralysis. Otherwise, a dystonia-predominant phenotype may also be developed. Neuroimaging studies have revealed atrophy in the cerebrum, brainstem, and spinal cord, as well as cerebral white matter lesions and a significant reduction in DAT uptake in the putamen. Given the diverse phenotypes observed in *COA7*-related disorders, in addition to CMT and SCD, it is recommended to include the *COA7* gene in disease-associated gene panels for various conditions, such as hereditary dystonia, familial Parkinson’s disease, leukoencephalopathy, and hereditary spastic paraplegia.

### Supplementary Information

Below is the link to the electronic supplementary material.

Supplementary file1 (PDF 899 KB)

## Data Availability

Data are available on request from the authors.
