# Supplementary material for: Dystonia and Parkinsonism in COA7-related disorders: expanding the phenotypic spectrum
Source: J Neurol. 2023 Sep 26;271(1):419–30. doi: 10.1007/s00415-023-11998-3 (PMC10769979; doi:10.1007/s00415-023-11998-3)
Supplement: Supplementary file 1 — Supplementary file1 (PDF 899 KB) [file 415_2023_11998_MOESM1_ESM.pdf]

*Journal of Neurology*
**Original Communication**

**Dystonia and Parkinsonism in COA7-Related Disorders: Expanding the Phenotypic Spectrum**

**Author information**

Yujiro Higuchi, MD, PhD[1)], Masahiro Ando, MD, PhD[1)], Fumikazu Kojima, MD [1)], Junhui Yuan, MD, PhD[1)], Akihiro Hashiguchi, MD, PhD[1)], Akiko Yoshimura, PhD[1)], Yu Hiramatsu, MD, PhD[1)], Satoshi Nozuma, MD, PhD[1)], Shinobu Fukumura, MD, PhD[2)], MD, PhD, Hiroyuki Yahikozawa, MD, PhD[3)], Erika Abe, MD, PhD[4)], Itaru Toyoshima, MD, PhD[4)], Masashiro Sugawara, MD, PhD[5)], Yuji Okamoto, MD, PhD[1)6)], Eiji Matsuura, MD, PhD[1)], and Hiroshi Takashima, MD, PhD[1)*].

1) Department of Neurology and Geriatrics, Kagoshima University Graduate School of Medical and Dental Sciences, Kagoshima, Japan
2) Department of Pediatrics, Sapporo Medical University School of Medicine, Sapporo, Japan
3) Yahikozawa Internal Medicine and Neurology Clinic, Nagano, Japan
4) Department of Neurology, National Hospital Organization Akita National Hospital, Yurihonjo, Japan
5) Department of Neurology, Akita University Graduate School of Medicine, Akita, Japan
6) Department of Physical Therapy, School of Health Sciences, Faculty of Medicine, Kagoshima University, Kagoshima, Japan

**Corresponding author:** Hiroshi Takashima, Email: thiroshi@m3.kufm.kagoshima-u.ac.jp

# Supplementary Information

## Supplementary figure 1: Conservation analysis

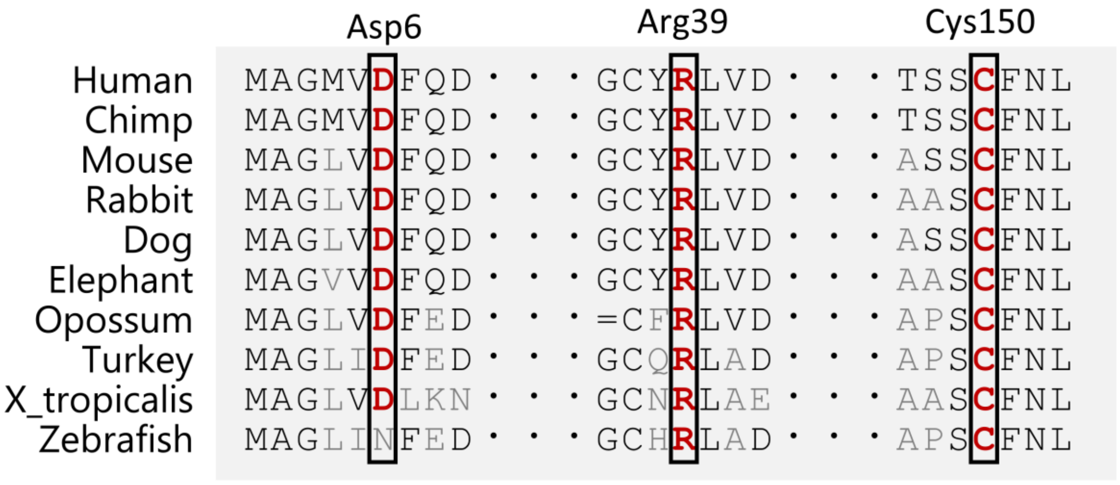


Comparison of *COA7* across different species. Asparatic acid 6, Arginine 39 and Cystein 150 are highly conserved among listed species.

## Supplementary figure 2: Evaluation of the structural model of COA7 mutant p.Cys150Tyr by SWISS-MODEL.

### Results

The SWISS-MODEL template library was searched for evolutionary related structures matching the target sequence in Table T1. For details on the template search, see Materials and Methods. Overall 1 templates were found (Table T2).

### Models

The following model was built (see Materials and Methods "Model Building"):

| Model #01 | File | Built with | Oligo-State | Ligands | GMQE | QMEANDisCo Global |
|---|---|---|---|---|---|---|
| 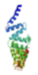 | PDB | ProMod3 3.2.1 | monomer | None | 0.97 | 0.91 ± 0.06 |

| Template | Seq Identity | Oligo-state | QSQE | Found by | Method | Resolution | Seq Similarity | Range | Coverage | Description |
|---|---|---|---|---|---|---|---|---|---|---|
| 7mqz.1.A | 99.52 | monomer | - | USER ALIGNMENT | X-ray | 2.39Å | 0.63 | 1 - 209 | 1.00 | Cytochrome c oxidase assembly factor 7 |

**The template contained no ligands.**

```
Target     EEQVKSFLENMEVECNYHCYHEKDPDGCYRLVDYLEGIRKNFDEAAKVLKFNCEENQHSDSCYKLGAYYVTGKGGLTQDL
7mqz.1.A   EEQVKSFLENMEVECNYHCYHEKDPDGCYRLVDYLEGIRKNFDEAAKVLKFNCEENQHSDSCYKLGAYYVTGKGGLTQDL

Target     KAAARCFLMACEKPGKKSIAACHNVGLLAHDGQVNEDGQPDLGKARDYYTRACDGGYTSSYFNLSAMFLQGAPGFPKDMD
7mqz.1.A   KAAARCFLMACEKPGKKSIAACHNVGLLAHDGQVNEDGQPDLGKARDYYTRACDGGYTSSCFNLSAMFLQGAPGFPKDMD

Target     LACKYSMKACDLGHIWACANASRMYKLGDGVDKDEAKAEVLKNRAQQLH
7mqz.1.A   LACKYSMKACDLGHIWACANASRMYKLGDGVDKDEAKAEVLKNRAQQLH
```

The quality of the structural model generated by SWISS-MODEL is evaluated using the QMEANDisCo Global score, which is 0.91±0.06 (recommended threshold: 0.5 or higher), indicating that the constructed model met the reliability criteria for modelling.

**Supplementary figure 3: Disulfide bond and position of Cys150 on the three-dimensional structure of human COA7**

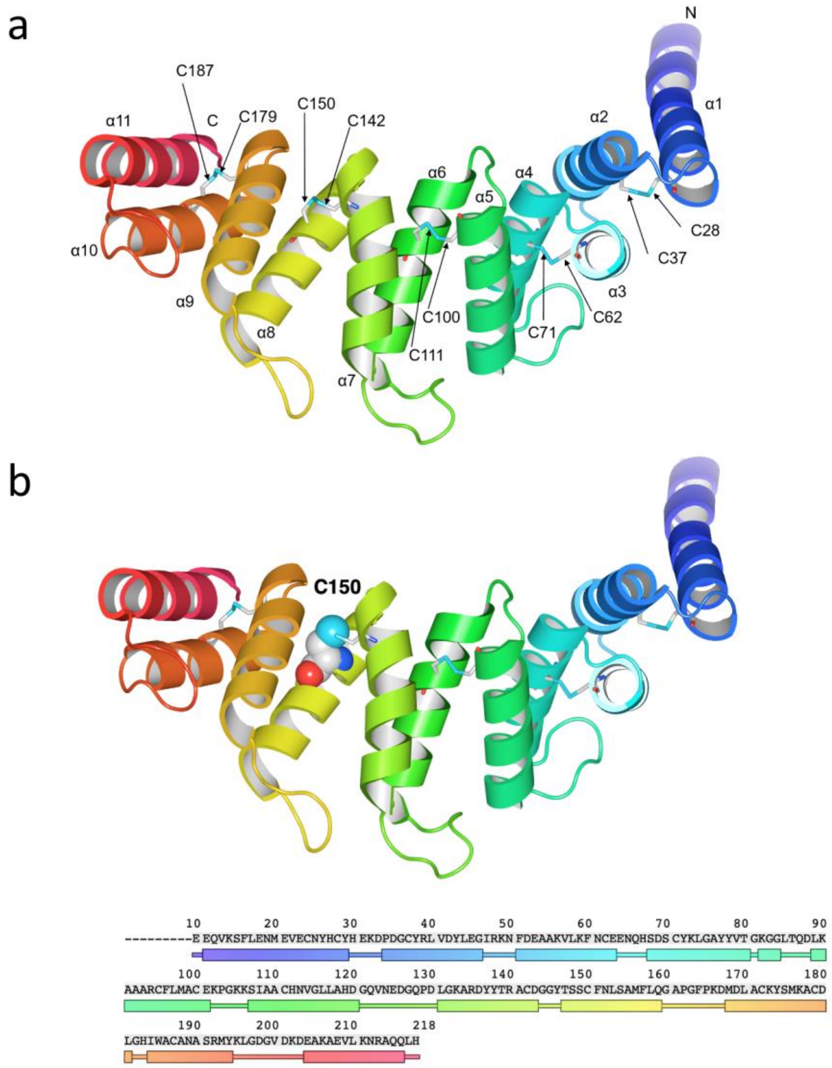


The three-dimensional structure of COA7 (PDB ID: 7MQZ) is shown in ribbon format with a blue-to-red gradient from the N-terminus to the C-terminus. The cysteine residues involved in disulfide bond formation are depicted in stick format, with carbon atoms in gray, oxygen atoms in red, nitrogen atoms in blue, and sulfur atoms in cyan. Figure a shows the positions of the disulfide bonds, while Figure b displays Cys150 in a space-filling model (top) and the amino acid sequence (bottom). Human COA7 is composed of 11 alpha helices (α1-α11), including five helix-turn-helix repeats (α/α repeats) and a single alpha helix at the C-terminus. Each of the 13 cysteine residues in COA7, of which 10 form disulfide bonds (Cys28-Cys37, Cys62-Cys71, Cys100-Cys111, Cys142-Cys150, Cys179-Cys187) between alpha helices in the α/α repeats. Supplementary Figure 3b shows the position of Cys150 in the three-dimensional structure of COA7. Cys150 is located in alpha helix 8 of the fourth α/α repeat (repeat_4) of COA7 and forms a disulfide bond with Cys142 in alpha helix 7 of the same repeat_4.

**Supplementary figure 4: COA7 expression data in the central nervous system obtained from the Human Protein Atlas project**

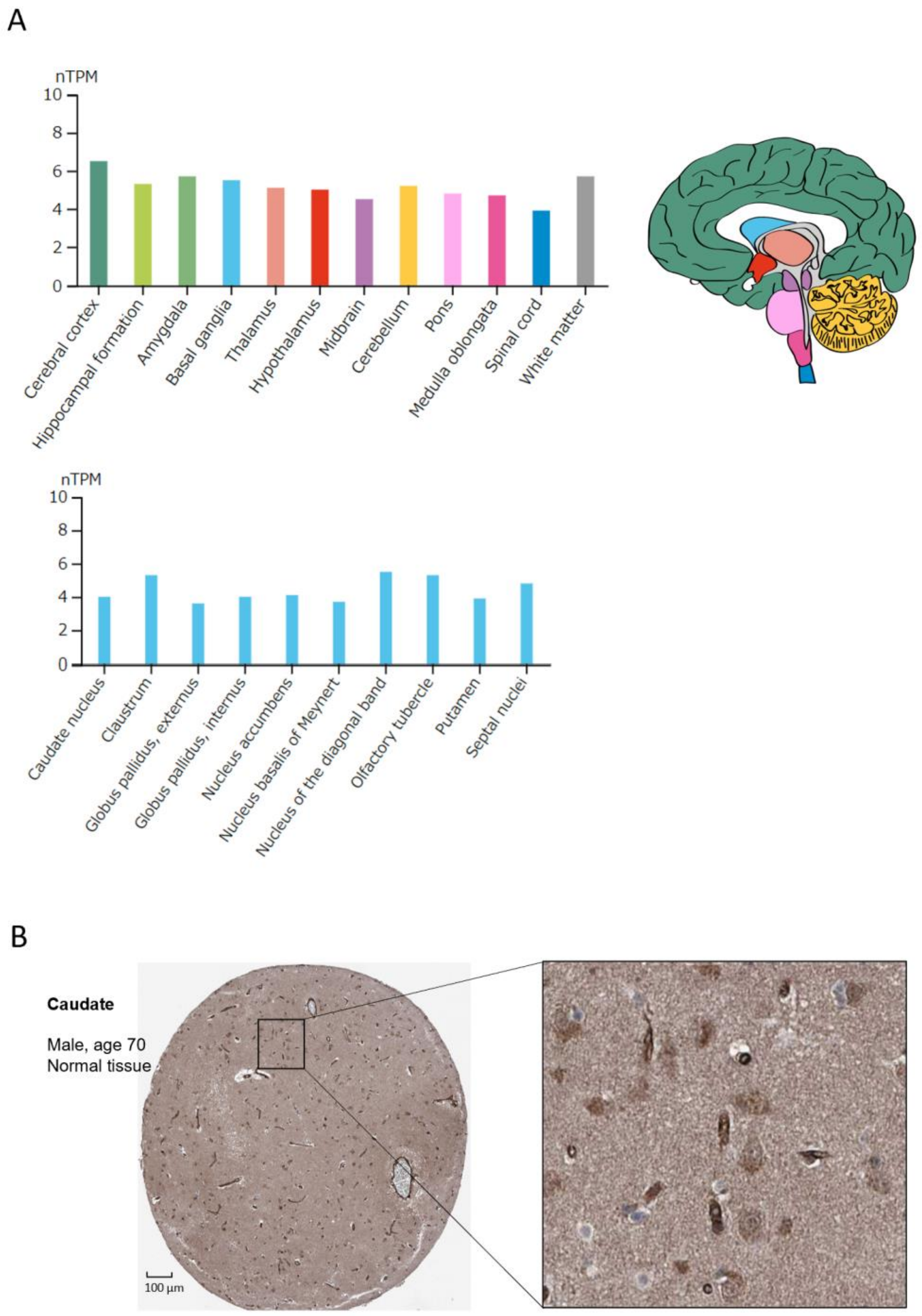


(A) Normalized RNA expression levels (nTPM) of COA7 in brain. RNA expression is widely observed in the brain including cerebral cortex, cerebellum, brain stem, basal ganglia and spinal

cord. It is also expressed in caudate nucleus, globus pallidus and putamen. RNA expression summary shows the consensus data based on normalized expression (nTPM) values from two different sources: internally generated Human Protein Atlas (HPA) RNA-seq data and RNA-seq data from the Genotype-Tissue Expression (GTEx) project. Color-coding is based on tissue groups, each consisting of tissues with functional features in common.

(B) Immunohistochemical (IH) staining with an anti-COA7 antibody (HPA028154) shows moderate expression of COA7 (brown) in neuronal cells in the caudate, but low in glial cells. Human Protein Atlas project: http://www.proteinatlas.org

**Supplementary table 1: Targeted CMT/IPN-related gene panels for DNA microarray and NGS.**

| | | | | | | | | |
|---|---|---|---|---|---|---|---|---|
| Microarray Chip sequencing (28 genes) | *AARS1* | *APTX* | *DNM2* | *EGR2* | *GAN1* | *GARS* | *GDAP1* | *GJB1* |
| | *HSPB1* | *HSPB8* | *LITAF* | *LMNA* | *LMNA* | *MFN2* | *MPZ* | *MTMR2* |
| | *NDRG1* | *NEFL* | *PMP22* | *PRX* | *RAB7* | *SBF2* | *SETX* | *SH3TC2* |
| | *SLC12A6* | *SOX10* | *TDP1* | *YARS1* | | | | |

| | | | | | | | | |
|---|---|---|---|---|---|---|---|---|
| 1st CMT gene panel (60 genes) | *AARS1* | *APTX* | *ARHGEF10* | *DHH* | *DNM2* | *EGR2* | *FGD4* | *FIG4* |
| | *GAN* | *GARS* | *GDAP1* | *GJB1* | *HARS* | *HK1* | *HOXD10* | *HSPB1* |
| | *HSPB8* | *KARS* | *LITAF* | *LMNA* | *MARS* | *MED25* | *MFN2* | *MPZ* |
| | *MTMR2* | *NDRG1* | *NEFL* | *PMP22* | *PRPS1* | *PRX* | *RAB7A* | *SBF2* |
| | *SETX* | *SH3TC2* | *SLC12A6* | *SOX10* | *TDP1* | *TRPV4* | *TTR* | *YARS* |
| | *20 candidate gene* | | | | | | | |

| | | | | | | | | |
|---|---|---|---|---|---|---|---|---|
| 2nd CMT gene panel (72 genes) | *AARS* | *APTX* | *ARHGEF10* | *COA7* | *DHH* | *DNM2* | *EGR2* | *FGD4* |
| | *FIG4* | *GAN* | *GARS* | *GDAP1* | *GJB1* | *HARS* | *HK1* | *HOXD10* |
| | *HSPB1* | *HSPB8* | *KARS* | *LITAF* | *LMNA* | *MARS* | *MED25* | *MFN2* |
| | *MPZ* | *MTMR2* | *NDRG1* | *NEFL* | *PMP22* | *PRPS1* | *PRX* | *RAB7A* |
| | *SBF2* | *SETX* | *SH3TC2* | *SLC12A6* | *SOX10* | *TDP1* | *TRPV4* | *TTR* |
| | *YARS* | *BSCL2* | *DCTN1* | *DHTKD1* | *DYNC1H1* | *FBLN5* | *FBXO38* | *GJB3* |
| | *GNB4* | *HSPB3* | *IGHMBP2* | *INF2* | *KIF1A* | *LRSAM1* | *PDK3* | *REEP1* |
| | *SBF1* | *SLC5A7* | *TFG* | *TRIM2* | *DCAF8* | *MME* | *SURF1* | *SACS* |
| | *GALC* | *PLEKHG5* | *6 Candidate genes* | | | | | |

| | | | | | | | | |
|---|---|---|---|---|---|---|---|---|
| 3rd CMT gene panel (103 genes) | *AARS1* | *AIFM1* | *ATP1A1* | *BSCL2* | *COX6A1* | *DCTN1* | *DHTKD1* | *DYNC1H1* |
| | *EGR2* | *FGD4* | *FIG4* | *GARS1* | *GDAP1* | *GNB4* | *HSPB1* | *HSPB3* |
| | *HSPB8* | *IGHMBP2* | *INF2* | *KARS1* | *LITAF* | *LMNA* | *LRSAM1* | *MARS1* |
| | *MCM3AP* | *MFN2* | *MME* | *MORC2* | *MPV17* | *MPZ* | *MTMR2* | *NAGLU* |
| | *NDRG1* | *NEFH* | *NEFL* | *PDK3* | *PLEKHG5* | *PMP2* | *PMP22* | *PNKP* |
| | *PRPS1* | *PRX* | *RAB7A* | *REEP1* | *SBF1* | *SBF2* | *SH3TC2* | *SIGMAR1* |
| | *SLC25A46* | *SLC5A7* | *SORD* | *SPG11* | *SURF1* | *VCP* | *WARS1* | *YARS1* |
| | *APTX* | *ARHGEF10* | *COA7* | *DNM2* | *FBLN5* | *GALC* | *GAN* | *GJB1* |
| | *GJB3* | *HARS1* | *HOXD10* | *KIF1A* | *SACS* | *SETX* | *SLC12A6* | *TDP1* |
| | *TFG* | *TRPV4* | *TTR* | *BAG3* | *DRP2* | *HINT1* | *LMNA* | *PDXK* |
| | *POLG* | *SCO2* | *ABHD12* | *SLC52A3* | *ATL1* | *ATL3* | *DNMT1* | *NTRK1* |
| | *PRDM12* | *SCN11A* | *SCN9A* | *SPTLC1* | *SPTLC2* | *WNK1* | *BICD2* | *DNAJB2* |
| | *LAMA2* | *GNE* | *SOD1* | *FUS* | *3 Candidate genes* | | | |

**Supplementary table 2: Prediction scores and allele frequency of detected variants in the *COA7* gene**

| Mutation | | c.17A>G | c.115C>T | c.449G>A |
|---|---|---|---|---|
| Protein | | p.Asp6Gly | p.Arg39Trp | p.Cys150Tyr |
| Position (GRCh38) | | chr1:52698310 | chr1:52692859 | chr1:52687967 |
| Reference | | T | G | G |
| Alternate | | C | A | A |
| dbSNP ID | | rs780572767 | rs768084335 | None |
| Prediction scores | SIFT | 0.014 (damaging) | 0.029 (deleterious) | 0 (Damaging) |
| | Polyphen2 | 0.86 (possible damaging) | 1.00 (probably damaging) | 0.325 (benign) |
| | PROVEAN | -3.71(damaging) | -4.51(Damaging) | -10.61(Damaging) |
| | MutationTaster | 1 (disease causing) | 0.9999 (disease causing) | 1 (disease causing) |
| | CADD (PHRED score) | 23.7 (deleterious) | 27.5 (deleterious) | 26.1 (deleterious) |
| MAF | jMorp (ToMMo 38KJPN) | C=0.000542 (44/77,444) homozygous count = 0 | C=0.000103 (8/77,444) homozygous count = 0 | NE |
| | ExAC | C=0.000008 (1/120,268) homozygous count = 0 | NE | NE |
| | gnomAD | C=0.000004 (1/248,356) homozygous count = 0 | C=0.000004 (1/251398) homozygous count = 0 | NE |

CADD = Combined Annotation Dependent Depletion (https://cadd.gs.washington.edu/snv); ESP = Exome Sequencing Project database (http://evs.gs.washington.edu/EVS/); jMorp: https://jmorp.megabank.tohoku.ac.jp/; ExAC = Exome Aggregation Consortium database (http://exac.broadinstitute.org/); HGVD = The Human Genetic Variation Database; MAF = minor allele frequency; NE = not exist; PROVEAN = Protein Variation Effect Analyzer; SIFT = Sorting Intolerant from Tolerant;

**Supplementary table 3: Electrophysiological findings of patients carrying biallelic variants in *COA7* in current and previous reports)**

| Patient No. | | | This study | | | Our past cases[1] | | | | Case by Anabel et al[2] | Case by Ban et al[3] | Case by Ouchi et al[4] |
|---|---|---|---|---|---|---|---|---|---|---|---|---|
| | | | Patient 1 | Patient 2 | Patient 3 | Patient 4 | Patient 5 | Patient 6 | Patient 7 | Patient 8 | Patient 9 | Patient 10 |
| Age at examination | | | 8 | 76 | 39 | 62 | 15 | 26 | 23 | n.a | n.a | 60 |
| Nerve | Median | DL | 2.5 | 3.6 | n.a | 3.9 | 3.1 | 3.8 | 3.6 | n.a | n.a | Normal in both limbs |
| | | MCV | 50.9 | 54.7 | 58.2 | 50.5 | 50.0 | 49.6 | 50.5 | n.a | n.a | |
| | | dCMAP | 17.1 | 5.1 | 1.8 | 8.0 | 4 | 4.7 | 14.0 | n.a | n.a | |
| | | SCV | ND | 45.8 | ND | ND | ND | ND | ND | n.a | n.a | |
| | | SNAP | ND | 2.0 | ND | ND | ND | ND | ND | n.a | n.a | |
| | Ulnar | DL | NE | 2.8 | n.a | 3.0 | NA | 2.9 | n.a | n.a | n.a | |
| | | MCV | NE | 66.7 | n.a | 61.8 | 56.5 | 52.8 | n.a | n.a | n.a | |
| | | dCMAP | NE | 4.4 | n.a | 7.4 | NA | 9.0 | n.a | n.a | n.a | |
| | | SCV | NE | 59.1 | ND | ND | NA | ND | ND | n.a | n.a | |
| | | SNAP | NE | 0.6 | ND | ND | NA | ND | ND | n.a | n.a | |
| | Tibial | DL | 4.1 | ND | ND | ND | ND | ND | n.a | n.a | n.a | n.a |
| | | MCV | 45.8 | ND | ND | ND | ND | ND | n.a | n.a | n.a | 33.9 |
| | | dCMAP | 9.86 | ND | ND | ND | ND | ND | 1.5 | n.a | n.a | 0.26 |
| | Peroneal | DL | NE | NE | n.a | NE | ND | n.a | n.a | n.a | n.a | ND |
| | | MCV | NE | NE | n.a | NE | ND | n.a | n.a | n.a | n.a | ND |
| | | dCMAP | NE | NE | n.a | NE | ND | n.a | n.a | n.a | n.a | ND |
| | Sural | SCV | ND | ND | ND | ND | ND | ND | ND | n.a | n.a | ND |
| | | SNAP | ND | ND | ND | ND | ND | ND | ND | n.a | n.a | ND |

DL: distal latency (ms); MCV: motor conduction velocity (m/s); dCMAP: distal compound muscle action potential (mV); SCV: sensory conduction velocity (m/s); SNAP: sensory nerve action potential (μV); n.a: not available; ND: not detected (evoked); NE: not examined


1) Higuchi Y, Okunushi R, Hara T, Hashiguchi A, Yuan J, Yoshimura A, et al. Mutations in COA7 cause spinocerebellar ataxia with axonal neuropathy. Brain. 2018;141:1622-36.
2) Martinez Lyons A, Ardissone A, Reyes A, Robinson AJ, Moroni I, Ghezzi D, et al. COA7 (C1orf163/RESA1) mutations associated with mitochondrial leukoencephalopathy and cytochrome c oxidase deficiency. J Med Genet. 2016.
3) Ban R, Liu Z, Shimura M, Tong X, Wang J, Yang L, et al. Biallelic COA7-Variants Leading to Developmental Regression With Progressive Spasticity and Brain Atrophy in a Chinese Patient. Front Genet. 2021;12:685035.
4) Ouchi S, Ishii K, Kosaki K, Suzuki H, Yamada M, Takenouchi T, et al. Parkinsonism in spinocerebellar ataxia with axonal neuropathy caused by adult-onset COA7 variants: a case report. BMC Neurol. 2023;23:211.

## Supplementary table 4: Mitochondrial and nuclear genes associated with dystonia and parkinsonism

| Gene Symbol | Gene/Locus MIM number | Phenotype MIM number | Phenotype | Function |
|---|---|---|---|---|
| *C19orf12* | 614297 | 614298 | Neurodegeneration with brain iron accumulation 4 | mitochondrial iron metabolism |
| *CARS2* | 612800 | 616672 | Combined oxidative phosphorylation deficiency 27 | Aminoacyl-tRNA synthetase |
| *CHCHD2* | 616244 | 616710 | Parkinson disease 22 | destabilization of cytochrome c |
| *CLPB* | 616254 | 619813 | Neutropenia, severe congenital, 9, autosomal dominant | cellular stress and heat shock responses, chaperone |
| *COA7* | 615623 | 618387 | Spinocerebellar ataxia, autosomal recessive, with axonal neuropathy | assembly of mitochondrial complex IV, heme-binding protein with disulfide reductase activity |
| *COASY* | 609855 | 615643 | Neurodegeneration with Brain Iron Accumulation 6 (NBIA6) | biosynthesis of coenzyme A |
| *COX20* | 614698 | 619054 | Mitochondrial complex IV deficiency, nuclear type 11 | assembly of mitochondrial complex IV |
| *COXFA4* | 603833 | 619065 | Mitochondrial complex IV deficiency, nuclear type 21? | subunit of complex IV (COX) |
| *DNAJC30* | 618202 | 619382 | Leber hereditary optic neuropathy, autosomal recessive | regulator of mitochondrial respiration, ATP synthase complex, chaperone protein, turnover of the subunits of mitochondrial complex I N-module. |
| *ECHS1D* | 602292 | 616277 | Mitochondrial short-chain enoyl-CoA hydratase 1 deficiency, Leigh-like, dystonia | Mitochondrial short-chain enoyl-CoA hydratase 1 |
| *FTL* | 134790 | 606159 | Neurodegeneration with brain iron accumulation 3 | mitochondrial iron metabolism |
| *HTRA2* | 606441 | 610297 | Parkinson disease 13, Encephalopathy + Dystonia | serine protease、apotosis |
| *LIPT1* | 610284 | 616299 | Lipoyltransferase 1 deficiency | lipid transfer between the cell membrane and mitochondria, fatty acid metabolism |
| *LIPT2* | 617659 | 617668 | Encephalopathy, neonatal severe, with lactic acidosis and brain abnormalities | lipid metabolism, transfer of phospholipids between the ER and mitochondria, |
| *LRPPRC* | 607544 | 220111 | Mitochondrial complex IV deficiency, nuclear type 5 | cytoskeletal organization, vesicular transport, transcriptional regulation of both nuclear and mitochondrial genes. |
| *LRRK2* | 609007 | 607060 | Parkinson syndrome 8 | serine/threonine-protein kinase, phosphorylation |
| *MECR* | 608205 | 617282 | Dystonia, childhood-onset, with optic atrophy and basal ganglia abnormalities | mitochondrial fatty acid synthesis |
| *MT-ND1* | 516000 | - | Alzheimer's/Parkinsonism, Leber hereditary optic neuropathy | mitochondrial complex I subunit |
| *MT-ND4* | 516003 | - | Spastic dystonia, Leber hereditary optic neuropathy | subunit of complex I |

| | | | | |
|---|---|---|---|---|
| *MT-ND6* | 516006 | - | Leigh with Complex I deficiency, Dystonia, deafness | subunit of complex I |
| *MT-TK* | 590060 | - | Parkinson syndrome, Neuropathy & Myopathy | mitochondrial tRNA for Lysine |
| *MT-TP* | 590075 | 545000 | Myopathy, MERRF, parkinsonism | mitochondrial tRNA for Proline |
| *MT-TT* | 590090 | - | Occasional sporadic Parkinsonism | mitochondrial tRNA for Threonine |
| *NDUFA12* | 614530 | 618244 | Mitochondrial complex I deficiency, nuclear type 23 | subunit of complex I |
| *NDUFA2* | 602137 | 618235 | Mitochondrial complex I deficiency, nuclear type 13 | subunit of complex I |
| *NDUFA9* | 603834 | 618247 | Mitochondrial complex I deficiency, nuclear type 26 | subunit of complex I, assembly of complex IV |
| *NDUFAF6* | 612392 | 618239 | Mitochondrial complex I deficiency, nuclear type 17 | assembly and maturation of complex I |
| *NDUFS1* | 157655 | 618226 | Mitochondrial complex I deficiency, nuclear type 5 | subunit of complex I |
| *NDUFS3* | 603846 | 618230 | Mitochondrial complex I deficiency, nuclear type 8 | subunit of complex I |
| *NDUFS6* | 603848 | 618232 | Mitochondrial complex I deficiency, nuclear type 9 | mitochondrial complex I subunit |
| *PANK2* | 606157 | 234200 | Neurodegeneration with brain iron accumulation 1 | coenzyme A biosynthesis |
| *PARK7* | 602533 | 606324 | Parkinson disease 7 | cell protection against oxidative stress and cell death acting as oxidative stress sensor and redox-sensitive chaperone and protease |
| *PINK1* | 608309 | 605909 | Parkinson syndrome 6 (Recessive; Early onset) | serine/threonine-protein kinase, mitochondrial quality control |
| *PNPT1* | 610316 | 614932 | Combined oxidative phosphorylation deficiency 13 | mitochondrial DNA replication |
| *PRKN* | 602544 | 600116 | Parkinson disease, juvenile, type 2 | component of a multiprotein E3 ubiquitin ligase complex, mitochondrial quality control |
| *SCO2* | 604272 | 604377 | Mitochondrial complex IV deficiency, nuclear type 2 | assembly of mitochondrial complex IV, synthesis and maturation of cytochrome c oxidase subunit II |
| *SERAC1* | 614725 | 614739 | 3-methylglutaconic aciduria with deafness, encephalopathy, and Leigh-like syndrome | regulation of mitochondrial phospholipid metabolism and the maintenance of mitochondrial membrane structure and function. |
| *SUCLG1* | 611224 | 245400 | Mitochondrial DNA depletion syndrome 9 | subunit of succinyl CoA synthetase, TCA cycle |
| *SURF1* | 185620 | 220110 | Mitochondrial complex IV deficiency, nuclear type 1 | cytochrome c assembly factor |
| *TACO1* | 612958 | 619052 | Mitochondrial complex IV deficiency, nuclear type 8 | mitochondrial translational activator. |
| *TIMM8A* | 300356 | 304700 | Deafness-Dystonia-Dementia syndromes, Mohr-Tranebjaerg syndrome | metabolite transporters from cytoplasm to mitochondrial inner membrane |
| *TWNK* | 606075 | 609286 | Progressive external ophthalmoplegia with mitochondrial DNA deletions, autosomal dominant 3 | mtDNA Helicase, mtDNA maintenance |
| *TXN2* | 609063 | 616811 | ?Combined oxidative phosphorylation deficiency 29 | maintaining the proper folding and function of many mitochondrial proteins by reducing disulfide bonds |
| *WARS2* | 604733 | 619738 | Parkinsonism-dystonia 3, childhood-onset | Aminoacyl-tRNA synthetase |
